# α-Mangostin Exhibits a Therapeutic Effect on Spinal Cystic Echinococcosis by Affecting Glutamine Metabolism

**DOI:** 10.1128/aac.00098-23

**Published:** 2023-05-04

**Authors:** Sibo Wang, Haohao Sun, Shan Wang, Qian Ren, Yi Dai, Meipeng Zhu, Yayun Zhang, Honglei Kang, Jing Li, Jun Xiao, Yimin Dong, Weishan Wang

**Affiliations:** a Department of Orthopedics, Tongji Hospital, Tongji Medical College, Huazhong University of Science and Technology, Wuhan, China; b The First Affiliated Hospital of Shihezi University, Shihezi City, Xinjiang Uygur Autonomous Region, China

**Keywords:** spinal echinococcosis, α-mangostin, glutamine, autophagy, anti-echinococcosis

## Abstract

Spinal cystic echinococcosis, a severely neglected, rare disease, is characterized by high morbidity, disability, and mortality in prevalent regions. Due to the high-risk nature of surgical treatment and the ineffectiveness of conventional drugs, there is an unmet need for novel safe and effective drugs for the treatment of this disease. In this study, we examined the therapeutic effects of α-mangostin for spinal cystic echinococcosis, and explored its potential pharmacological mechanism. The repurposed drug exhibited a potent *in vitro* protoscolicidal effect and significantly inhibited the evolution of larval encystation. Moreover, it demonstrated a remarkable anti-spinal cystic echinococcosis effect in gerbil models. Mechanistically, we found that α-mangostin intervention led to intracellular depolarization of mitochondrial membrane potential and reactive oxygen species generation. In addition, we observed elevated expression of autophagic proteins, aggregation of autophagic lysosomes, activated autophagic flux, and disrupted larval microstructure in protoscoleces. Further metabolite profiling showed that glutamine was imperative for autophagic activation and anti-echinococcal effects mediated by α-mangostin. These results suggest that α-mangostin is a potentially valuable therapeutic option against spinal cystic echinococcosis through its effect on glutamine metabolism.

## INTRODUCTION

Cystic echinococcosis (CE) is a cosmopolitan parasitic zoonosis, caused by the larvae of *Echinococcus granulosus* (*E. granulosus*) parasites in humans and other mammals. Osseous hydatidosis occurs in 0.5% to 4% of all cases (1), the spine being the most commonly involved part of the skeleton in approximately 50% of cases (2). The prognosis of spinal CE is similar to that of malignancies: a mortality rate of >50% has been reported within 5 years after symptom onset (3–5). In spinal CE, the parasite infiltrates and spreads along the trabeculae of the bones and can invade the vertebral pedicle and laminae, showing obvious pathological “osteolytic” manifestations and causing pathological fractures. Some hydatid cysts can also invade the surrounding soft tissue to form paravertebral and intraspinal cysts (6). The specific molecular mechanism of bone destruction caused by hydatid tissue is still unclear and more studies are needed to uncover its potential mechanisms to aid drug development.

Since Reydellet performed the first such treatment in 1819, surgery has remained the main therapeutic option in spinal CE (1). It includes simple drainage or debridement, scraping of the lesion, and excision of the infected bone. The principle of surgery is a radical resection with a safety margin of 2 cm, which is rarely possible in patients with vertebral diseases (1). However, for some patients who have missed the best time for surgical treatment, pharmacological treatment is the best option. Two drugs, albendazole (ABZ) and mebendazole (MBZ), are clinically preferred for conservative treatment; they inhibit the growth of echinococcal tissue by blocking the energy metabolism (7, 8). However, the efficacy of both drugs is poor in approximately 40% of patients due to the drawbacks of weak intestinal absorption and low blood levels (9, 10). Patients with spinal CE have even lower local blood concentrations of ABZ and MBZ, these drugs are even less efficacious in spinal-CE patients than in liver and lung CE patients due to the presence of a bony barrier at parasitization site (11). Moreover, exploratory research into the pharmacological treatment of CE has been limited to intra-abdominal parasites, while basic research on bone or spinal CE is still lacking. Therefore, it is clinically important to find new anti-spinal CE drugs and explore new molecular targets in the process of parasite metabolism to provide safer, more effective, noninvasive treatments for spinal CE.

Natural products are great prospects for the development and application of antiparasitic drugs due to their low toxicity and various biological activities (12). α-mangostin (α-MG) is a representative natural xanthone isolated from the pericarps of mangosteen. It has been proven to possess a variety of pharmacological properties, such as anticarcinogenic, anti-inflammatory, antidiabetic, and antioxidant activities (13, 14), as well as hepatoprotective, cardioprotective, and neuroprotective properties. Of these, its anticarcinogenic activity is the most promising (15–17). It is reported that α-MG significantly increased light chain 3 (LC-3) expression levels in chronic myeloid leukemia cells, suggesting that α-MG induces autophagy in cancer cells (17). In addition, α-MG can induce apoptosis in osteosarcoma cells through reactive oxygen species (ROS)-mediated endoplasmic reticulum stress via the *Wnt* pathway (18). Recently, the antiparasitic potential of α-MG has been gradually uncovered: the drug has been confirmed to inhibit Caenorhabditis elegans activity *in vitro*, similar to the effects exerted by MBZ (19). Moreover, the combination of α-MG with 9-hydroxycalabaxanthone demonstrated a synergistic antimalarial effect (20). However, the anti-CE effects of α-MG are still unclear. In this study, we aimed to investigate the pharmacodynamic role played by α-MG against spinal CE and the potential mechanism of the drug.

## RESULTS

### α-MG exhibited parasiticidal effect and cystic differentiation suppression against *E. granulosus* in the larval stages.

**FIG 1:**
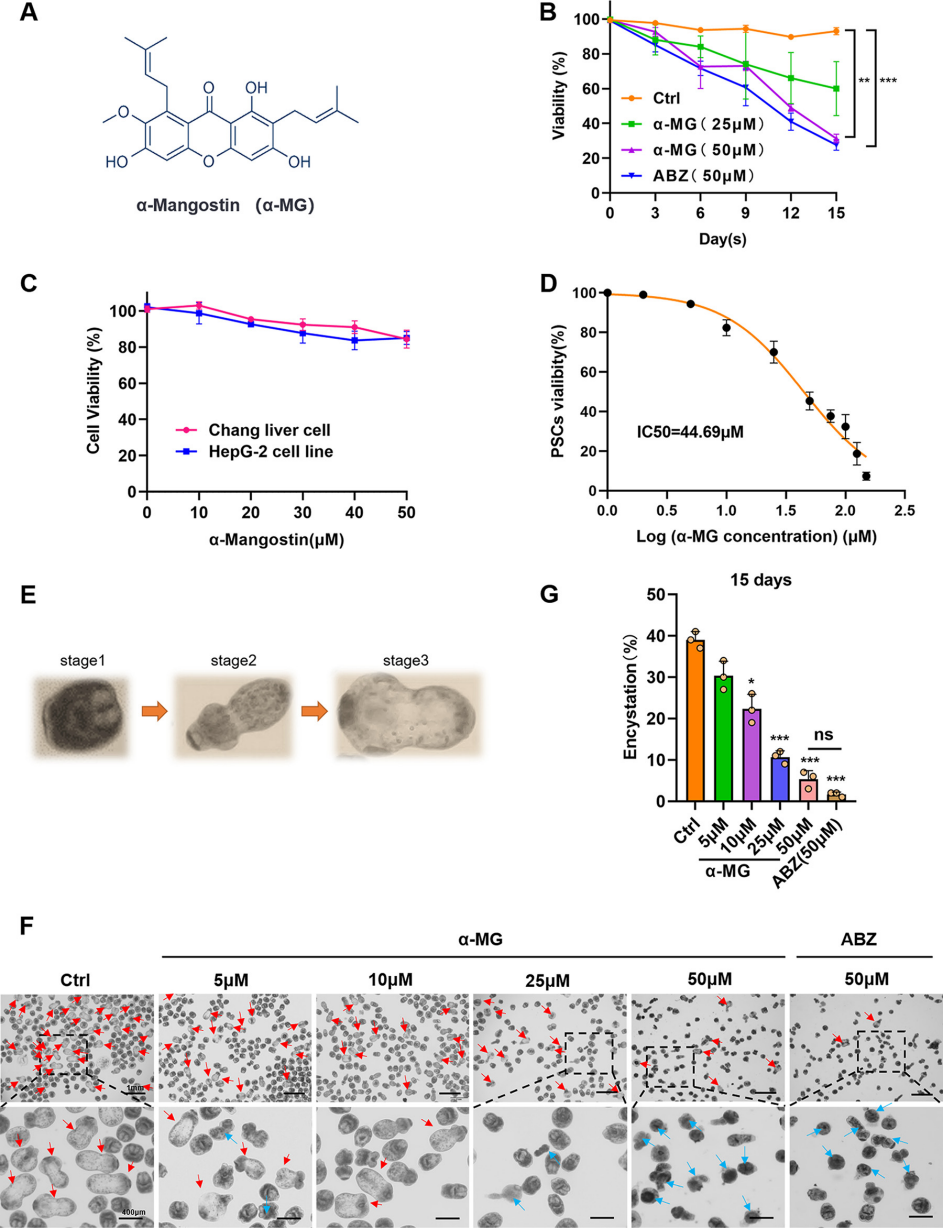
The effect of α-MG on *E. granulosus* PSCs cultured *in vitro*. (A) The structural formula of α-MG. (B) PSCs were cultured for 15 days *in vitro* in the presence of 25 µM, 50 µM α-MG, and 50 µM ABZ; parasites incubated in culture medium containing DMSO served as controls (Ctrl). Survival of PSCs was assessed every 24 h using the Eosin dye exclusion test (*n* = 3). (C) Cytotoxicity of α-MG was assessed by MTT in Chang liver cell line and HepG-2 cell line (*n* = 3). The cytotoxicity was not detected. (D) To estimate the half maximal inhibitory concentration (IC50), PSCs were incubated in 48-well plates at different concentrations (ranging from 1 μM to 150 μM) of α-MG. IC50 was calculated using a nonlinear regression equation. (E) Three distinct physiological stages experienced by hydatid larvae during *in vitro* evolution. (F) PSCs were cultured for 15 days to detect the evolution of encystation. Red arrows indicate encysted larvae, blue arrows indicate dead larvae. (G) α-MG markedly inhibited the encystation rate of PSCs, which is consistent with the effect of ABZ (*n* = 3). Data shown are represented as mean ± SD. of at least three independent experiments. **, *P* < 0.01; ***, *P* < 0.001 using the two-sided Student’s-*t* test.

To explore the *in vitro* protoscolicidal effect of α-MG, we established low-dose (25 µM) and high-dose (50 µM) groups for the treatment of Protoscoleces (PSCs) cultured *in vitro*. After 15 days of intervention, α-MG exhibited a significant parasiticidal effect, with a 39% ± 13% decrease in PSC viability in the low-dose group and a 69% ± 2% decrease in the high-dose group, which showed the same antiparasitic ability as ABZ (72 ± 2.5%, Fig. 1B). The biosafety of α-MG therapy was assessed using a methyl thiazolyl tetrazolium (MTT) assay (Fig. 1C). We found that the viability of both Chang and HepG2 cells lines were > 85% at the experimental dose. We then calculated the IC50 of α-MG as 44.69 µM (Fig. 1D).

Hydatid larvae go through three distinct physiological stages during CE development in an *in vitro* culture. With adequate culturing, the larvae gradually differentiate into the cystic state (Fig. 1E). Therefore, we treated cultured PSCs with different drug concentrations, and counted differentiated cystic larvae in various groups. The results showed that α-MG could significantly inhibit the encystation efficacy of PSCs. Most PSCs treated with α-MG were shrunken, accompanied by dissolution of the worm structure (Fig. 1F). Moreover, after 15 days of intervention, only 13% of PSCs differentiated into cysts in the 25 µM α-MG group, which was lower than 38% in the control group. Meanwhile, the minimum encystation rate in the 50-µM group was 5%, which was not significantly different from the effect of the same dose of ABZ (2%, Fig. 1G). In conclusion, at a safe dose, α-MG showed an antiparasitic effect, that was not inferior to that of the clinical first-line drug ABZ.

### α-MG induced reactive oxygen species and disrupted mitochondrial function in PSCs.

**FIG 2:**
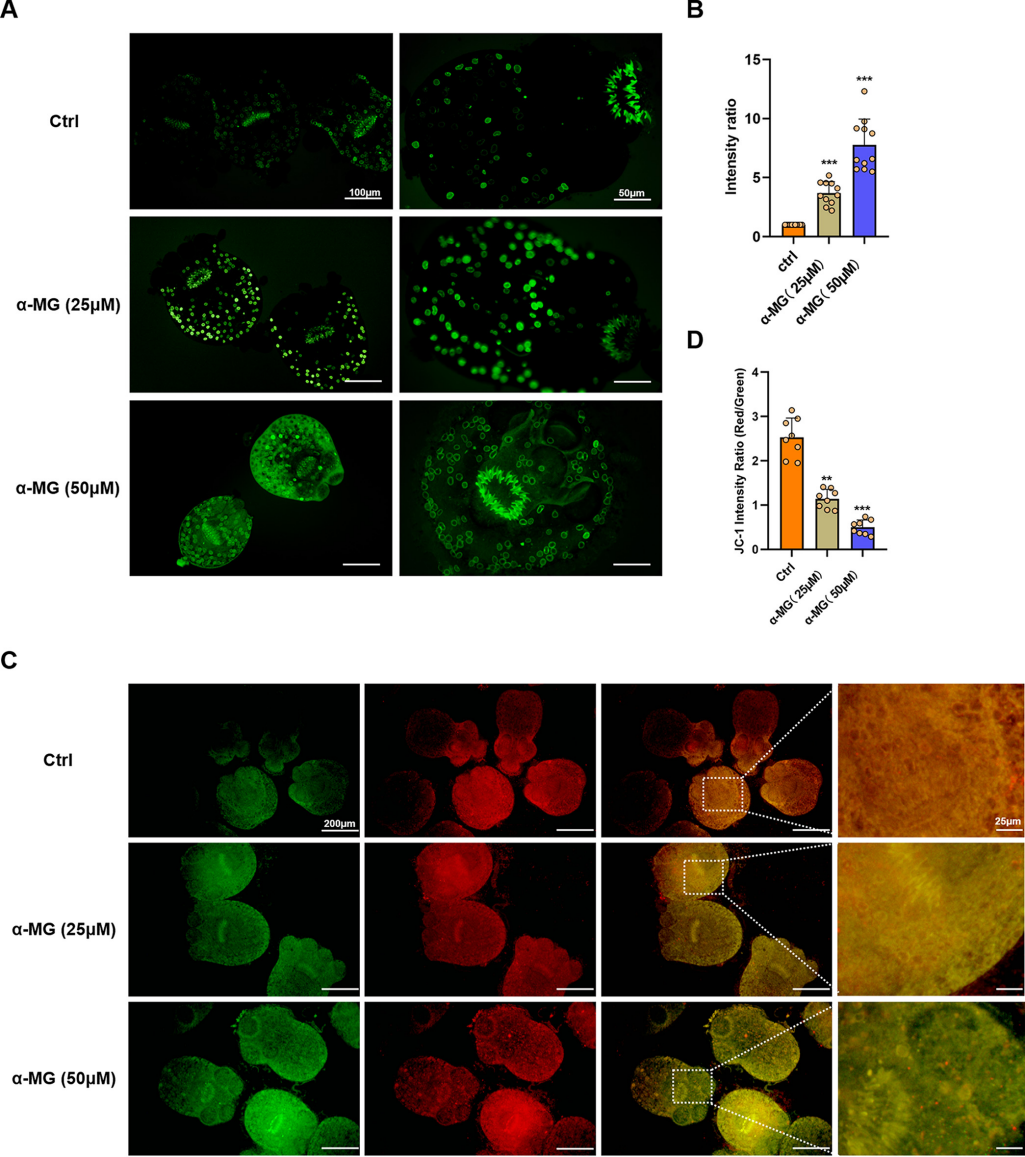
α-MG-induced ROS production and mitochondrial damage in PSCs. (A) Representative images showing ROS fluorescence in PSCs incubated 3 days with 25 µM, 50 µM α-MG. (B) ROS fluorescence intensity of PSCs in different treatment groups (*n* = 11). (C) JC-1 fluorescent staining of PSCs after 3 days treated with 25 µM, 50 µM α-MG. (D) The values of the red/green JC-1 fluorescence ratios were measured in different groups (*n* = 8). Data are presented as mean ± SD. **, *P* < 0.01; ***, *P* < 0.001 using the two-sided Student's *t* test.

To analyze the potential biological mechanism of α-MG, we performed an ROS assay on PSCs after drug intervention and found that α-MG increased ROS generation in PSCs (Fig. 2A and B). Then, we used the novel cationic carbocyanine dye JC-1 to label the treated PSCs. Interestingly, after α-MG intervention, intracellular mitochondrial membrane potential (MMP) of PSCs in the 25-µM treatment group showed depolarization, as indicated by the green fluorescence of JC-1 monomers (Fig. 2C and D). The effect was amplified in the high-dose treatment group (50 µM), a trend consistent with ROS level. This suggested that α-MG could induce the aggregation of ROS and disrupt mitochondrial function of PSCs.

### α-MG-induced ROS triggered autophagy in PSCs.

**FIG 3:**
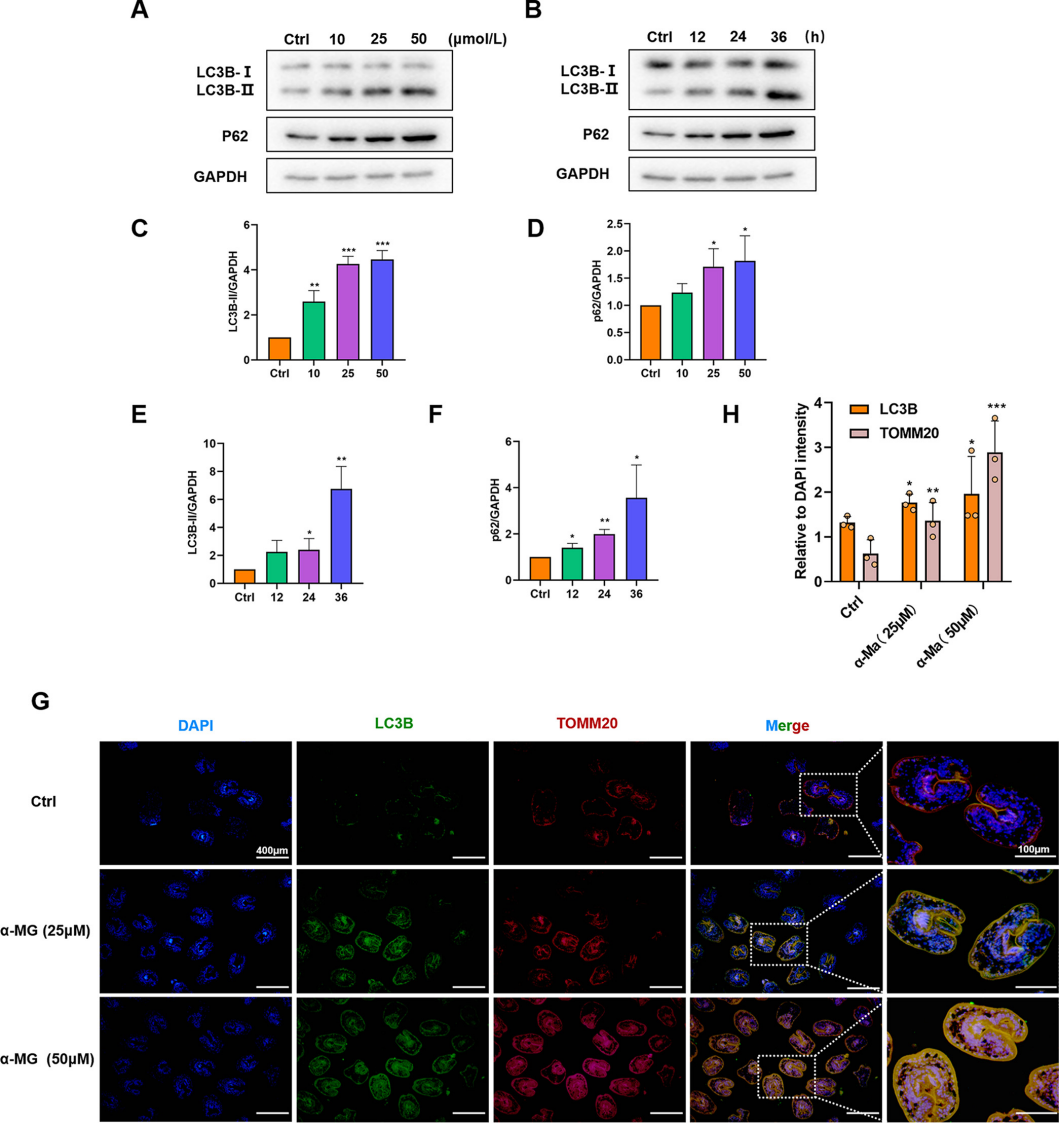
PSCs autophagy was induced by α-MG. (A) Representative Western blots of LC3B and p62 under the effect of gradient drug concentration. (B) Representative Western blots of LC3B and p62 under the time effect of α-MG. (C and D) Quantification of LC3B and p62 with the dose effect. (*n* = 3) (E and F) Quantification of LC3B and p62 with the time effect. (*n* = 3) (G) Representative fluorescence images of immunolocalization assays of PSCs in each group after 25 µM, 50 µM α-MG intervention. (H) Quantitation of LC3B and TOMM20 colocalization in different treatment groups. PSCs were incubated with α-MG for the indicated time, fixed, and stained with anti-LC3B and anti-TOMM20 to label target proteins (*n* = 3). Data are presented as mean ± SD. *, *P* < 0.05; **, *P* < 0.01; ***, *P* < 0.001 using the two-sided Student's *t* test.

It is generally known that the impairment of mitochondrial function is closely related to the occurrence of autophagy (21). Therefore, we examined the expression levels of autophagy-related proteins in PSCs that had been exposed to α-MG. Immunoblotting assay results showed that incubation with α-MG enhanced LC3B and p62 levels in PSCs, in a dose- and time-dependent manner (Fig. 3A to F). Moreover, we determined the colocalization (yellow) of TOMM20 (red) with autophagosome-localized LC3B (green) via immunofluorescence (IF) staining (Fig. 3G). The expression of both autophagy-related proteins increased significantly in drug-treated PSCs, and higher drug concentrations (50 µM) increased this effect (Fig. 3H). The above experiments indicated that α-MG treatment induced activation of autophagy in PSC.

To clarify the role of ROS production in PSCs autophagy, we used MitoQ10 to block oxidative stress under α-MG intervention. MitoQ10 as a mitochondrion-targeted antioxidant can block H_2_O_2_-induced intracellular ROS generation and prevent oxidative damage. We first verified the inhibitory effect of MitoQ10 on ROS production (Fig. S1A and B). Subsequent IF staining results showed that MitoQ10 effectively inhibited the expression of LC3B and TOMM20 proteins, suggesting that ROS was essential for induction of autophagy in PSCs. (Fig. S1C and D).

### α-MG disrupted protoscolex microstructure and promoted the development of autophagy.

**FIG 4:**
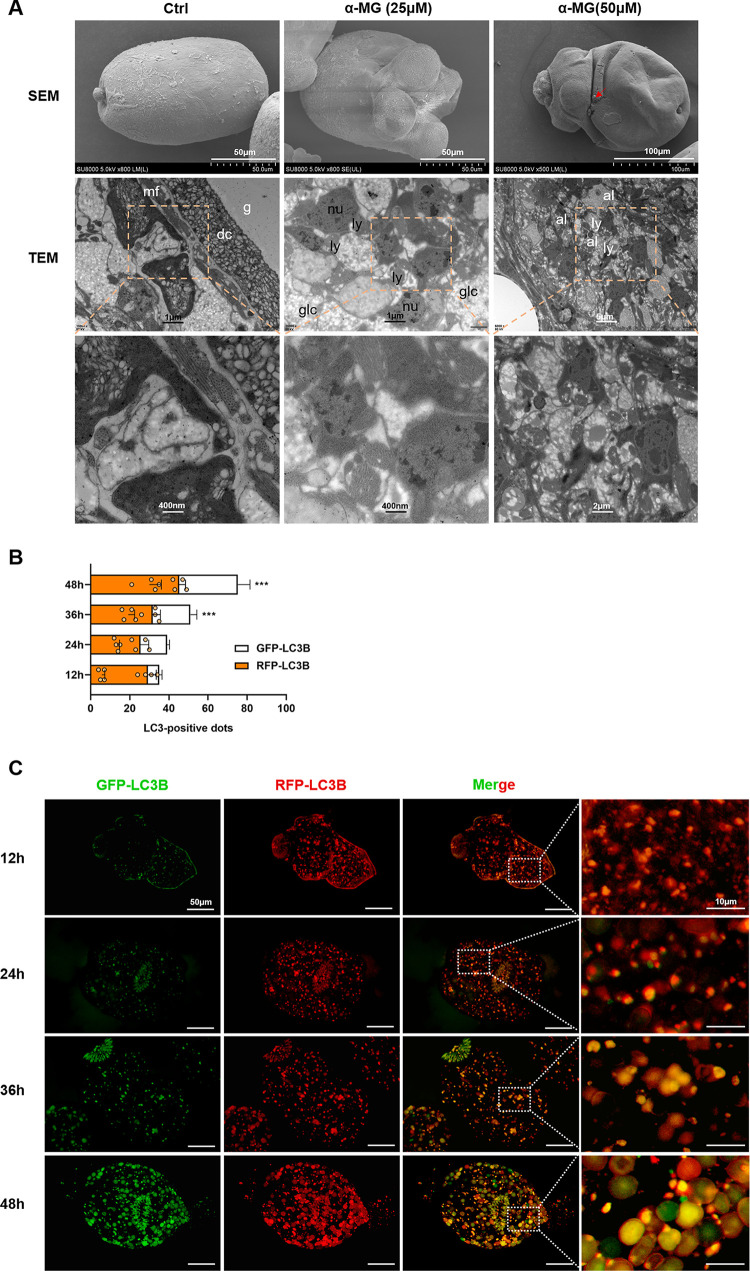
Ultrastructural changes and autophagic flow is triggered by α-MG in the larval stage of *E. granulosus.* (A) Representative SEM and TEM images of PSCs incubated under control conditions or with 25 µM, 50 µM α-MG for 3 days. In SEM, the PSCs in the low dose of α-MG groups presented some contraction of partial region. In the high-dose group, the PSCs ruptured and gradually dissolved as red arrow point. The control PSCs exhibited no ultrastructural alterations in parasite tissue. In TEM, a reduction in glycogen storage and some lysosomes were found in the low dose of α-MG group. PSCs in the high-dose group showed an abundant amount of autophagosomes and lysosomes with nucleolysis. glc, glycogen; g, glycocalyx; dc, distal cytoplasm; mf, muscular fibers; nu, nucleus; ly, lysosomes; al, autophagolysosomes. (B and C) Representative images and quantification of autophagosomes (yellow dots produced by overlapping GFP and RFP puncta) after 24 h treatment with α-MG, shown as yellow bars, and autophagosomes (red dots produced by RFP puncta), shown as red bars. Effect of 50 µM α-MG treatment in PSCs transfected with the Ad-mCherry-GFP-LC3 (*n* = 8). The results shown are means ± SD. ***, *P* < 0.001. Bars indicate: 50 µm and 10 µm.

To further understand the autophagy-based effectiveness of α-MG, we confirmed structural and ultrastructural changes in treated-PSCs via scanning electron microscopy (SEM) and transmission electron microscopy (TEM) (Fig. 4A). As observed under the TEM, the larval body was wrinkled after stimulation with by 25 µM α-MG. Notably, the soma region of PSCs in the high dose group (50 µM) was markedly altered, with fissures appearing in the tegument, which represented the beginning of bodily lysis. Moreover, TEM analysis of PSCs treated with 25 µM α-MG revealed glycogen consumption, tissue disorganization, and the presence of lysosomes. Additionally, we found large numbers of autophagosomes and lysosomes after treatment with 50 µM α-MG. To confirm this finding, we counted GFP-LC3B dots, which were a good indicator of autophagosomes and were closely correlated with the state of autophagic flux. As shown in Fig. 4C, 12 h after treatment with α-MG (50 µM), there was a marked increase in red dots. As treatment progressed, both yellow and red dots peaked after 48 h, suggesting that α-MG intervention led to autophagosomal accumulation and facilitated autophagic flux in PSCs (Fig. 4B). Collectively, these results indicated that α-MG could disrupt the tissue structure of parasites, induce autophagy in PSCs, and kill them.

### α-MG blocked the growth of parasitic cysts in gerbils with spinal cystic echinococcosis.

**FIG 5:**
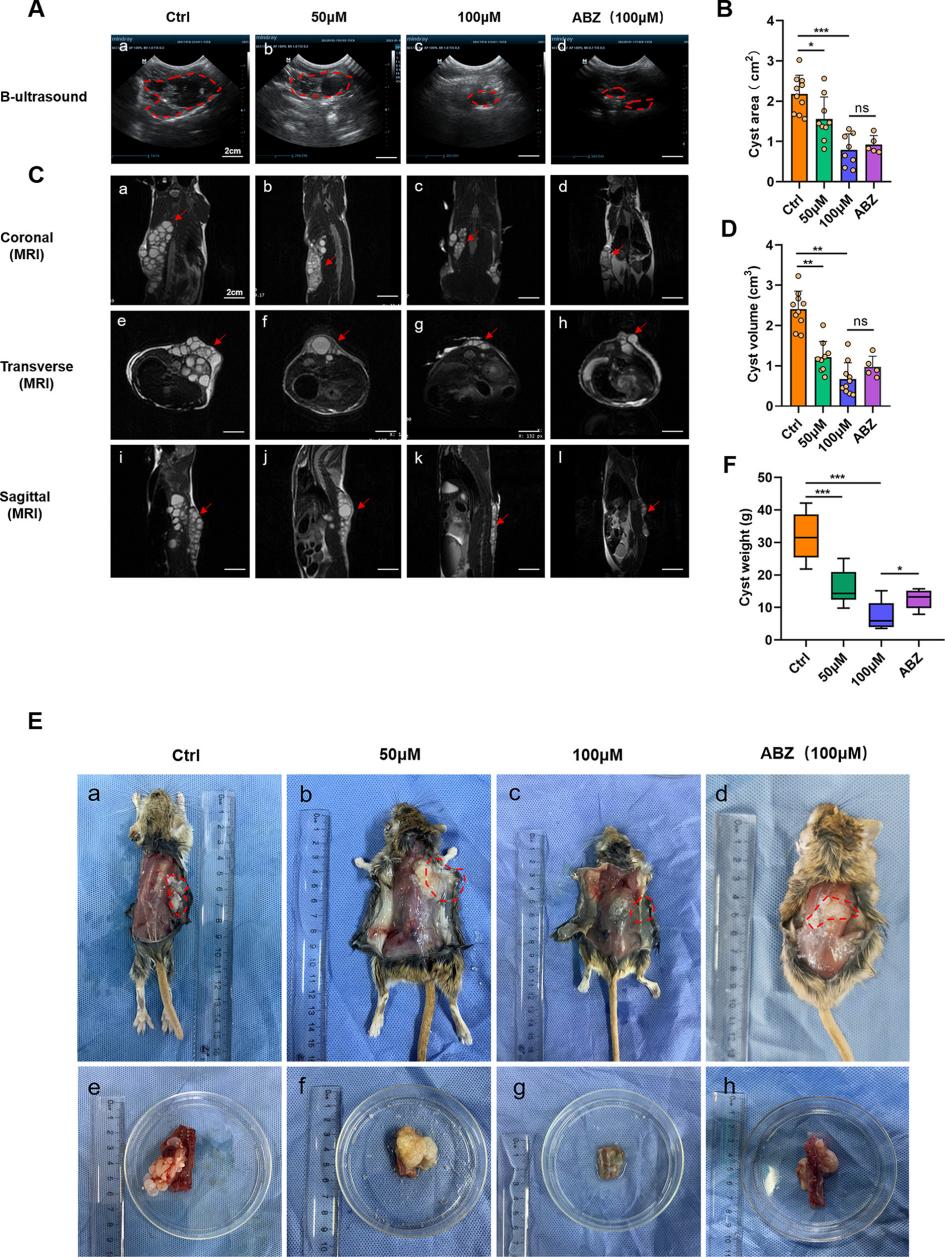
α-MG is effective against spinal CE in gerbils. (A) The gerbils were infected with 3,000 *E. granulosus* PSCs and intraperitoneal injection with 50 µM, 100 µM α-MG in different groups for 8 weeks (twice a week). A total of 100 µM ABZ was used as a positive control. Equal amount of solvent injection was used as control. The location, shape, boundary, and internal echo of abnormal echo focus, blood flow signal, as well as maximum lesion diameter were recorded by B-ultrasound. Clear image was reserved to calculate the lesion area (cm^2^). (B) The cross-sectional area of the cysts in the ultrasound images were calculated and compared. (*n* ≥ 5). (C) Images of sagittal, coronal plane, and transverse section were captured by MRI. Red arrow indicates CE tissue. (D) The volumes of cysts in MRI pictures were calculated and compared. (*n* ≥ 5). (E) Spinal hydatid tissue was isolated and the cysts were weighed after thorough dissection. (F) Weight of cysts in different groups were measured. (*n* ≥ 5). Results shown are represented as mean ± SD. *, *P* < 0.05; **, *P* < 0.01; ***, *P* < 0.001 using the two-sided Student’s-*t* test.

The *in vivo* effect of α-MG against *E. granulosus* cysts was tested in gerbils infected with cultured PSCs. After confirming the completion of spinal CE model construction by magnetic resonance imaging (MRI), we treated PSCs with α-MG. The cyst area as measured by small animal B-ultrasound at the end of the 8-week treatment period showed that 50 µM (1.56 ± 0.52 cm^2^) of α-MG had significant parasiticidal activity, compared with untreated controls (2.18 ± 0.44 cm^2^). Moreover, there was no significant difference in therapeutic effect between the 100-µM α-MG (0.79 ± 0.37 cm^2^) and the ABZ (0.92 ± 0.20 cm^2^) groups (Fig. 5A and B). In addition, MRI yielded consistent results for cyst volume (Fig. 5C). The high-dose (0.67 ± 0.38 cm^3^) group had the greatest reduction in parasitic burden, compared with the ABZ (0.97 ± 0.24 cm^3^), low-dose (1.2 ± 0.37 cm^3^), and control (2.4 ± 0.43 cm^3^) groups (Fig. 5D). To further clarify the efficacy of α-MG *in vivo*, we removed spinal CE tissue after treatment, and also excised the involved spinal segments at the same time. Finally, pure *E. granulosus* cysts were resected and weighed. Surprisingly, the 100-µM α-MG group (7.4 ± 4.0 g) showed the best antiparasitic effects, even stronger than those in the ABZ group (12.6 ± 2.7 g; Fig. 5F). In the control group, the spine was invaded by a large amount of cystic tissue, with multiple segments involved. In contrast, parasitic-cyst growth was almost completely inhibited in the high-dose group, with minimal involvement of spinal segments (Fig. 5E). These data fully demonstrated that intraperitoneal (i.p.) administration of α-MG showed a strong anti-echinococcosis effect *in vivo*.

**FIG 6:**
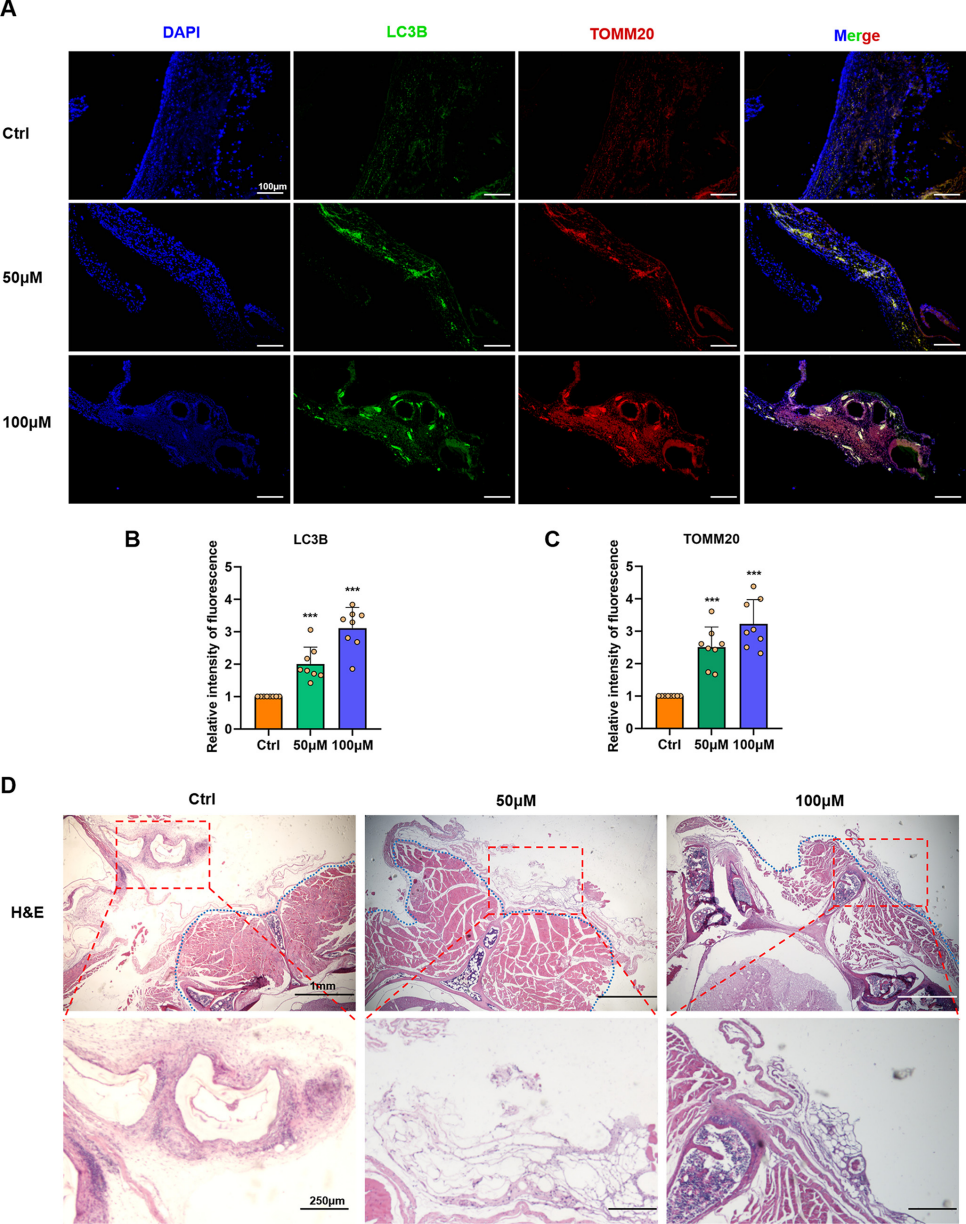
Drug-induced autophagy of *in vivo* echinococcal tissue and inhibition of spinal CE growth. (A) Representative IF staining images of spinal worm tissue. (B and C) Quantification of the autophagy protein fluorescence intensity in different treatment groups (*n* = 8). (D) HE staining of spinal hydatid tissue sections. The blue dotted line represents the boundary of normal spinal tissue. Data are the mean ± SD. ***, *P* < 0.001 using the two-sided Student’s-*t* test.

IF staining of spinal cysts showed that α-MG could significantly activate cell autophagy in echinococcal tissues with a prominent dose-dependent effect (Fig. 6A to C). Autophagic proteins were enriched in the laminar layer of the echinococcal cyst, as well as in blood vessels. Then, hematoxylin and eosin (H&E) staining of controls showed the spine and paravertebral muscles were heavily infested with CE tissue (Fig. 6D). Conversely, α-MG treatment strongly inhibited the growth of the parasitic tissue, with a significant reduction in echinococcal cyst size, and this effect was enhanced when the dose was high.

### The anti-echinococcal effect of α-MG was dependent on glutamine deprivation.

To explore potential pharmacological mechanism, we used principal component analysis (PCA) to clarify the natural distribution and discrimination of metabolites between PSCs given different treatments (Fig. SA). We then analyzed the data to find the difference in amino acid concentration in two groups (Table S1). A volcano plot showed that concentrations of Gln and Lys increased significantly after α-MG intervention, while Arg and Thr showed the most significant decreases in concentration (Fig. S2B). The results of Z-score cluster analysis between the two groups are shown in Fig. S2C, listing differential metabolites in PSCs. Therefore, we hypothesized that α-MG interfered with the amino acid metabolism of PSCs and thus exerted a protoscolicidal effect. We then selected two amino acids from the most significant differences in increasing and decreasing concentrations to validate the drug targets. To this end, PSCs were cultured in differently conditioned media. The results showed that simply adding or removing amino acids alone did not significantly affect PSC activity, at least not during short-term incubation. After α-MG intervention, PSCs in Gln-deficient medium showed significant activity reversion compared with the drug-only group. This result suggested that extracellular Gln was necessary for the antiparasitic effect of α-MG, and Gln metabolomics was a potential target for drug action (Fig. S2D). To strengthen this conclusion, we enabled encystation experiments to be performed under different culture and intervention conditions. Excitingly, we cross-enriched for Gln deficiency in multiple culture conditions affecting PSC differentiation *in vitro* (Fig. S2E). Inhibition of cystic differentiation by α-MG was reversed by Gln deficiency (Fig. S2F). Through these experiments we screened the targets of drug action and performed different assays to verify that Gln was essential for the effectiveness of the α-MG.

### Gln was responsible for inducing autophagy in PSCs.

**FIG 7:**
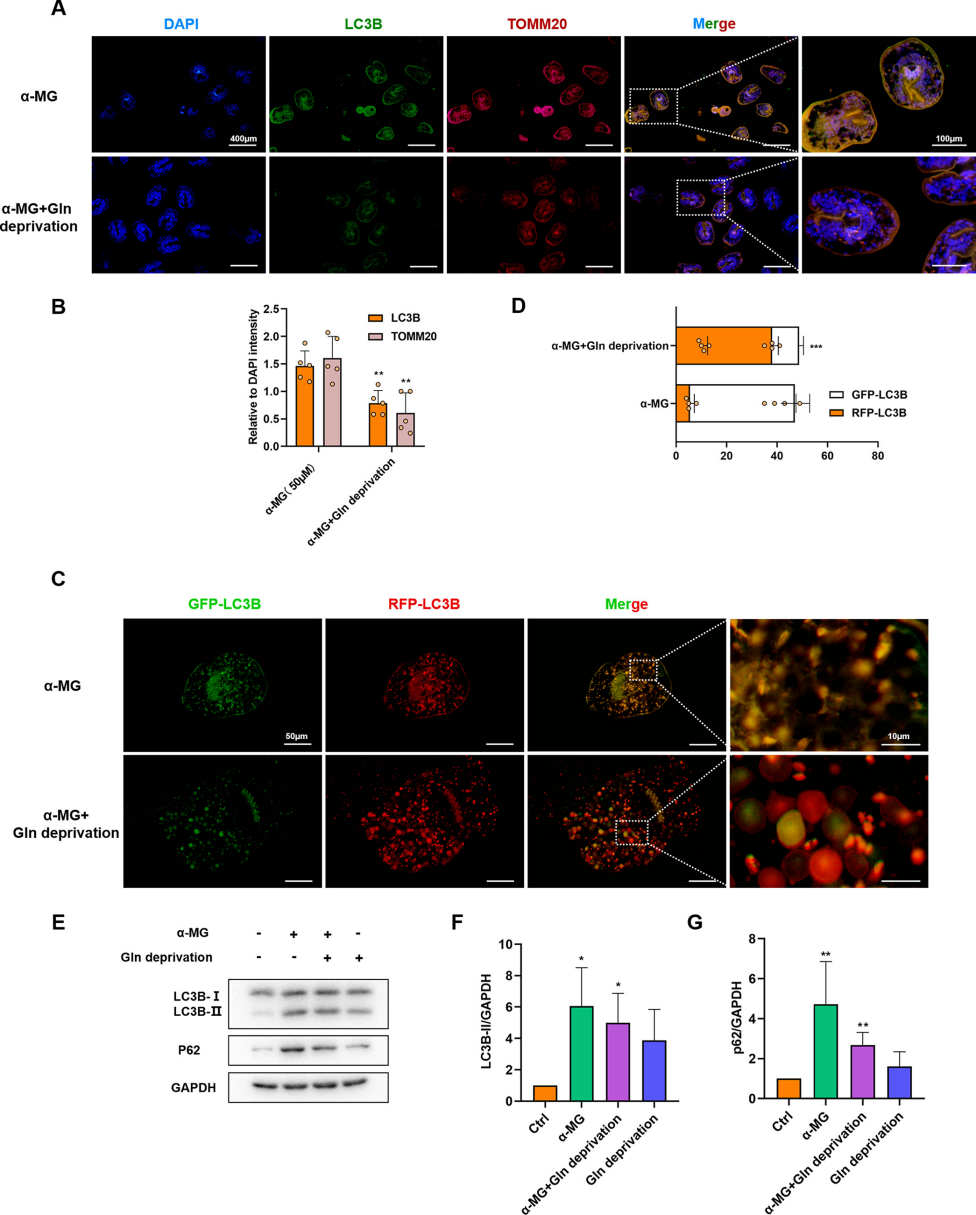
α-MG induces autophagy in PSCs cells via Gln. (A) Representative fluorescence images of immunolocalization assays of PSCs in glutamine deprivation conditioned medium after 50 µM α-MG intervention. (B) Quantification of LC-3 and TOMM20 co-localization in different conditioned medium groups (*n* = 5). PSCs were stained with anti-LC-3 and anti-TOMM20 to label target proteins after α-MG intervention. (C and D) Representative images and quantification of 50 µM α-MG-induced autophagosomes and autophagolysosomes after 24 h of treatment with glutamine-deficient conditioned medium (*n* = 8). (E) The expression of LC3B and p62 in PSCs was assessed by Western blot analysis. (F and G) Quantification of LC3B and p62 protein levels (*n* = 3). Data are presented as mean ± SD. *, *P* < 0.05; **, *P* < 0.01; ***, *P* < 0.001 using the two-sided Student’s-*t* test.

Because Gln was the *de facto* drug target, we activated autophagy in PSCs with α-MG in the presence and absence of Gln and performed IF to detect the expression levels of autophagic proteins. As shown in Fig. 7A to B, lack of Gln rescued the α-MG-induced autophagic proteins LC3B and TOMM20 in PSCs. Consistent with these results, the number of drug-induced GFP-LC3B dots was significantly reduced after Gln deprivation, implying that the process of autophagic flux was blunted (Fig. 7C to D). Moreover, the increase in LC3B and p62 protein expression levels induced by α-MG was largely blocked by Gln deficiency (Fig. 7E to G). Overall, these results, together with that of metabolites data, demonstrated that Gln was a key component of autophagic induction activated by α-MG, and also played a key role in the drug’s parasiticidal action.

### Toxicology of α-MG *in vivo*.

We evaluated the *in vivo* toxicity of α-MG via morphological observation of liver and kidney tissues *in vivo*. H&E-stained images showed no significant pathological changes or damage to these tissues (Fig. S3). In addition, liver and kidney function indices of gerbils after 2 months of treatment with effective concentrations of α-MG showed no significant changes compared with the control group (Table S2).

## DISCUSSION

Despite significant advances in diagnostic imaging techniques, surgical treatment, and pharmacological therapy over the past century, spinal CE continues to have high morbidity, disability, and mortality rates in prevalent regions (22). In particular, its recurrence rate of nearly 45% makes surgical treatment much less effective. ABZ, the only clinically licensed antiparasitic drug, has not been replaced by a new compound for nearly half a century since its introduction. Due to its considerable side effects, it is administered at inhibitory rather than parasiticidal concentrations (23, 24). Novel compounds are therefore urgently needed. α-MG is a main bioactive compound derived from the mangosteen fruit, with significant therapeutic potential against tumors, inflammation, metabolic disorders, and many other conditions (25). In recent studies, it has shown some antiparasitic activity, such as antimalarial effects in combination with chlorhexidine and anti-malarial effects in combination with 9-hydroxycalabaxanthone (20, 26). In this study, α-MG was demonstrated for the first time to have a strong anti-spinal CE effect.

To the best of our knowledge, PSCs evolve morphologically as cysts in a suitable culture environment over a long period. Their morphology is characterized by enlargement of the PSC’s head, expansion and transparency of the somites, gradual union of the rostellum, transformation of the whole body into a transparent oval or round shape, and decomposition of the calcium granules in larvae. This is the precursor state of microcysic production. The process of cyst formation is completed once the laminated and germinal layers are gradually formed, which is also the ultimate state of the worm after entering the intermediate host. Notably, the inhibitory effect of α-MG on PSCs viability was also captured in our encystation intervention experiments. The specific regulatory mechanism underlying larval differentiation is still unknown; it needs to be further explored in subsequent studies because it could be a potential target for precision treatment of anti-echinococcal disease.

Numerous studies have reported that α-MG exerts its biological effects through induction of ROS accumulation in target cells, such as induction of apoptosis in human osteosarcoma cells and human rheumatoid fibroblast-like synoviocytes, and inhibition of invasion of pancreatic cancer cells (18, 27, 28). To explore the underlying mechanisms of α-MG treatment of hydatids, we identified the facilitative effect of α-MG on ROS production in PSCs. Mitochondria are well known as important mediators of cellular metabolism processors and targets of ROS (29). Whether the mechanism of α-MG repression is similar to that of ABZ in that it blocks the parasite's uptake of multiple nutrients and glucose and inhibits the production of ATP, making it impossible for the parasite to survive and reproduce, needs further exploration (30).

Given the regulatory role of autophagy in the anti-tumor and anti-inflammatory effects of α-MG (31, 32), we examined the expression levels of autophagic proteins in PSCs after drug stimulation in terms of time and concentration, respectively. In addition to the temporal dosage effect, by fluorescence localization, we found that autophagic proteins were aggregated on the envelopes of PSCs and partially expressed on the suckers, which could reduce larval defense against the environment. Several studies in recent years have identified autophagy as a regulator of drug-induced death of *E. granulosus*. As a protease inhibitor, bortezomib exhibits potent deleterious effects in PSCs and metacestodes *in vitro*, and this antiparasitic effect is achieved by upregulating autophagy-related genes (Eg-atg6, Eg-atg8, Eg-atg12, and Eg-atg16) in larval stages of *E. granulosus* (33). In addition, metformin, the first-line drug in the treatment of type 2 diabetes, was recently discovered to have anti-echinococcal effects by blocking cellular energy metabolism via activation of the AMP-activated protein kinase (AMPK-) forkhead box protein O (FoxO) signaling pathway (34). These studies suggest that autophagy has potential as a regulatory target for drugs in studies exploring anti-CE compounds. It must be emphasized that this is the first attempt to characterize hydatid autophagic flux by counting GFP-LC3B dots in PSCs.

In a previous study, we established a gerbil model of spinal CE to enable *in vivo* evaluation and analysis of anti-hydatid treatment, and we established a detailed modeling method and a model-forming evaluation system that depended on multiple imaging examinations. Herein, 8 weeks after i.p. injection, we found significant inhibition of spinal-hydatid tissue growth. Interestingly, on MRI coronal view we found scoliosis, a common clinical spinal deformity, in gerbils in the control group. The cause of this deformity might be destabilization of the vertebral body by infestation of echinococcal tissue or a secondary deformity caused by the long-term pain-avoidance position of the model gerbils. The former is more likely based on the atypical clinical features of spinal CE symptoms (35, 36). Another point of special interest is that in the low-dose group we found significant calcification of echinococcal cyst tissues, which was in contrast to the transparent cysts in the control group. In the high-dose group, this phenomenon was not as pronounced due to the marked inhibition of cyst growth. This finding is encouraging because calcification of the cyst wall is considered a pathological feature of echinococcal tissue necrosis (37). In clinical management, imaging findings of massive spontaneous calcification of the cyst wall are an important principle for avoiding surgical intervention (38). Such calcification might be related to the host’s immune resistance or to infection by hydatid tissue. The related mechanisms, which scholars continue to study in depth, could be a new regulatory target for the future treatment of echinococcosis.

It has been reported that α-MG has a protective effect against Gln-induced cytotoxicity in HT22 hippocampal neurons (39). To explore the pharmacological mechanisms of α-MG, we sequenced the targeted metabolomics of PSCs after different treatments. Ultimately, Gln was found to be the key to drug effectiveness. CE is well known to lack the ability to synthesize pyrimidines, purines, and most amino acids (except for alanine, aspartic acid, and glutamic acid) *ab initio*. Gln is converted to glutamate by amino acid metabolism after ingestion by hydatids and collaborates with ketoglutarate to participate in the tricarboxylic acid cycle (TCA) to produce the energy required for metabolism (40). This may be the reason why α-MG influenced mitochondrial function and activates ROS production. Our results suggest that α-MG may alter energy production through alterations of the TCA in PSCs.

In conclusion, this drug repurposing study proved that α-MG exhibited considerable effects against both the larval- and adult-stages of spinal CE. α-MG activated ROS and induced autophagy-derived cell death by disrupting the mitochondrial function of *E. granulosus*, and these effects were achieved by impacting Gln metabolism. This naturally produced plant can therefore offer an alternative for the noninvasive treatment of spinal CE.

## MATERIALS AND METHODS

### Ethics statement.

We carried out all protocols involving animals in strict accordance with the *Guidelines for the Care and Use of Laboratory Animals* (8th ed.) The study was also approved by the Institutional Animal Care and Use Committee (IACUC) of the First Affiliated Hospital of Shihezi University (No. A2019-163-01). Surgery was performed under gas anesthesia, and best efforts were made to minimize unnecessary animal suffering.

**(i) Antibodies and reagents.** All antibodies were purchased from Abcam (Cambridge, UK). LC3B (#ab192890), p62 (#ab109012), translocase of outer mitochondrial membrane 20 (TOMM20; #ab186735), and secondary antibodies (#ab150077, #ab150113). We obtained mitoquinone (MitoQ10; #HY-100116A) mesylate from MedChemExpress (Monmouth Junction, NJ, USA). α-MG (#M3824) and ABZ (#54965-21-8) were purchased from Sigma-Aldrich (St. Louis, MO, USA).

### Parasites, animals, and infection.

**(i) Isolation of parasites.** PSCs were acquired and cultured as previously described (41).

**(ii) Spinal CE models.** Male Mongolian gerbils (weight, 50 to 80 g; aged, 6 to 8 weeks) were purchased from the Experimental Animal Center of Xinjiang Medical University (Urumqi, Xinjiang; Experimental Animal License No. SCXK 2020-0005). The employed methods for parasitic infection and animal anesthesia were previously described in detail (41).

### Viability test.

After 3 days of culture, we collected PSCs and washed them several times in phosphate-buffered saline (PBS). In pharmacodynamic testing, approximately 3,000 PSCs were seeded onto 24-well plates and divided into the following four groups: control group (with identical amounts of dimethyl sulfoxide [DMSO] and drug solvent), a 25-µM/L α-MG, a 50 µM/L α-MG, and a 50 µM/L ABZ group. To detect essential amino acids for drug targeting, PSCs were treated under 10 different conditions. We established arginine (Arg; #74-79-3; Sigma-Aldrich), threonine (Thr; #6028-28-0; Acmec Biochemical Co., Ltd.; Shanghai, China), glutamine (Gln)-deficient (#10372019; Invitrogen Corp., Carlsbad, CA, USA), and lysine (Lys)-deficient conditioned medium (#YC-2067, Shanghai Yuchun Biotechnology Co., Ltd., Shanghai, China) group, with Roswell Park Memorial Institute (RPMI) 1640 medium (Thermo Fisher Scientific; MA, USA) as control. The other five groups had the same amino acid conditions combined with α-MG intervention (50 µM/L). To prepare Arg and Thr conditioned medium, we added 5 mM/L of each amino acid’s reserve liquid to RPMI 1640 medium and mixed them at room temperature (RT) for 5 min. An eosin dye (#G1120; Solarbio; Beijing, China) exclusion test was used for viability analysis. Pharmacological intervention lasted 15 days, with medium changes every 2 days. We calculated the half-maximal inhibitory concentration (IC50) of α-MG via nonlinear regression using GraphPad Prism software (8th; GraphPad Software, Inc., San Diego, CA, USA).

### Cystic differentiation efficacy.

To evaluate the pharmaceutical efficacy of α-MG, we incubated 3,000 PSCs in 24-well plates in the presence of increasing concentrations (5 to 50 µM) of α-MG until day 15. For controls, we used medium with identical amounts of DMSO and ABZ (50 µM). To explore drug-targeted amino acids, PSCs were divided into 10 groups as described above. We performed an encystation assay, changing the medium every 2 days. Percentages of encysted PSCs were estimated by counting a minimum of 200 PSCs per well.

### ROS and mitochondrial function assays.

**(i) ROS.** Before staining, PSCs in the three groups (0, 25, and 50 µM α-MG) were cultured for 72 h after different treatments. We added 10 µM MitoQ10 to the 50 µM α-MG group to inhibit the production of ROS. After three washes with PBS, intracellular ROS levels were detected using a fluorescent probe with dichlorofluorescin diacetate (DCF-DA; #ab113851; Abcam). We stained PSCs with 10 µM DCF-DA for 45 min at 37°C in the dark. After three more washes, fluorescence images were collected under excitation at 540 nm and emission at 590 nm under a fluorescence microscope (IX83; Olympus; Tokyo, Japan), and ROS levels were detected using ImageJ software 2.1 (National Institutes of Health [NIH], Bethesda, MD, USA).

**(iii) Mitochondrial membrane potential.** JC-1 (#T4069; Life Technologies, [Invitrogen], Carlsbad, CA, USA) is an ideal fluorescent probe for evaluating levels of MMP change. The relative ratio of red to green fluorescence is used to assess the extent of mitochondrial depolarization and PSC viability. We cultured the three groups of PSCs (0, 25, and 50 µM α-MG) for 72 h under different treatments and stained them with JC-1 as described previously (41).

### Western blot analysis.

We divided PSCs into four groups and treated them with different doses of α-MG (0, 10, 25, and 50 µM) for 24 h. To assess the drug’s effects over time, PSCs were treated with 50 µM α-MG for 0, 12, 24, and 36 h. After intervention, we lysed PSCs with radioimmunoprecipitation assay (RIPA) buffer (#R0278; Sigma-Aldrich) and crushed them ultrasonically. After centrifugation (13,000 r/min, 4°C, 10 min), protein supernatants were isolated from the lysate for subsequent analysis. We separated polypeptides via by sodium dodecyl sulfate-polyacrylamide gel electrophoresis (SDS-PAGE) on 10% polyacrylamide gels and then electrophoretically transferred them to polyvinylidene fluoride (PVDF) membranes. Membranes (MilliporeSigma, Burlington, MA, USA) were blocked with 5% skimmed milk at RT for 2 h, and then incubated with LC3B and p62 primary antibody (1:1,000) overnight at 4°C. After washing them three times in tris-buffered saline with Tween (TBST), we incubated the membranes with corresponding secondary antibodies (1:20,000) for 1 h in RT. An Enhanced Chemiluminescence Kit (Thermo Fisher Scientific, Waltham, MA, USA) was used to detect bands.

### Autophagy analysis.

**(i) IF.** After treating with different doses of α-MG (0, 25 and 50 µM) for 24 h, we rinsed PSCs three times with PBS. Samples were fixed in 10% buffered formalin phosphate (24 h), dehydrated, and embedded in paraffin; the blocks were cut into at 4-µm thick sections. After deparaffinizing and rehydrating them, we rinsed slides with PBS and incubated them with blocking buffer (1% bovine serum albumin [BSA] in PBS) for 30 min. Sections were incubated overnight at 4°C with antibodies against LC3B (1:200) and TOMM20 (1:200) proteins. After three additional washed in PBS, slides were incubated with secondary antibodies. We subjected the sections to analysis under an Olympus Immunofluorescence Laser Scanning Confocal Microscope.

**(ii) Measurement of fluorescent LC3B puncta.** PSCs were cultured on 24-well plates and then transiently transfected with Ad-mCherry-Green Fluorescent Protein (GFP-) LC3B (#C3011; HanBio Technology, Shanghai, China) per manufacturer’s instructions. After 24 h, we treated the PSCs with different doses of α-MG as described above. Next, PSCs were washed three times with PBS and then fixed with 4% formaldehyde for 15 min at RT. We observed LC3B puncta under the Olympus confocal microscope. Numbers of mCherry and GFP dots were determined by manual counting of fluorescent puncta.

### Ultrastructural analysis via SEM and TEM.

Control and treated PSCs (25, 50 µM for 24 h) were processed for SEM (JEM1200EX, JEOL Ltd., Japan) and TEM as previously described (41). We used SEM to observe the general morphology and structural integrity of the PSCs and TEM to observe the formation of autophagic bodies and autophagic lysosomes.

### *In vivo* efficacy of α-MG against spinal CE.

After 4 months of infection with spinal CE, all gerbils were confirmed by MRI to be modeled. We divided them into high-dose (100 µM) and low-dose (50 µM) treatment groups according to the intervention protocol and administered drugs to them via i.p. injection under gas anesthesia. We used 100 µM ABZ as a positive control. The control group was injected with the same dose of vehicle solution. We administered α-MG twice a week for 8 weeks. After treatment, all gerbils were examined by MRI and B-ultrasound to clarify and quantify the sizes and locations of spinal CE lesions. The gerbils were subsequently euthanized, and parasitic tissue was removed for weighing. Specific parameters of the imaging setup and method were described previously (41).

### Histological and IF assays.

**(i) H&E.** Spinal CE samples were fixed with 4% paraformaldehyde (PFA) for 48 h, sequentially dehydrated, embedded in paraffin, and sectioned (at a thickness of 5 µm). We then stained the sections with H&E stains.

**(ii) IF.** Sections were deparaffinized with xylene and then rehydrated with ethanol. We performed antigen retrieval using citrate buffer (0.1 M, pH 6.0). After sealing by QuickBlock Blocking Buffer (#P0260, Beyotime, Institute of Biotechnology, Shanghai, China) for 1 h, we added Triton 100 (#85111; Sigma-Aldrich). Subsequently, sections were incubated overnight (4°C) with primary antibodies (LC3B and TOMM20, 1:200). Then, we probed the slides with anti-mouse/rabbit Alexa Fluor 488 or 568 secondary antibodies for 1 h and labeled them with 4′,6-diamidino-2-phenylindole (DAPI; #D9542; Sigma-Aldrich). Finally, we examined the sections under the confocal microscope and evaluated them using ImageJ software.

### Liver and kidney toxicity of α-MG *in vivo*.

We collected peripheral blood from gerbils in the high-dose treatment and control groups to detect serum biochemical indicators such as total bilirubin and alkaline phosphatase (ALP). Afterward, all livers and kidneys were collected and immersed in 10% formalin before histopathological examination.

### Metabolic quantification analysis.

PSCs cultured *in vitro* were divided into a drug treatment group (50 µM α-MG) and a control group. After 72 h of intervention, PSCs were thoroughly crushed under ultrasound and the supernatant was extracted after centrifugation. To fully precipitate the proteins and extract the metabolites, 1 mL of RIPA buffer was added per sample. Liquid nitrogen was snap-frozen, sonicated, and incubated at low temperature for more than 1 h. Next, the supernatant was lyophilized and 100 μL of sodium chloride was added. After dissolution, the supernatant was extracted again and the samples were sent to NOVOGENE Co., Ltd. (Beijing, China) for metabolite extraction and liquid chromatography-tandem mass spectrometry (LC-MS) analysis. We used Student's *t* test for statistical analysis. Metabolites with variable importance in projection (VIP) > 1, *P < *0.05, and fold change (FC) > 2 were considered differential metabolites.

### Statistical analysis.

All data in experiments were presented as mean ± standard deviation (SD) from at least three independent experiments, and significance was determined using Student's *t* test or a nonparametric Kruskal-Wallis test, as appropriate. One-way ANOVA was chosen for the comparison between multiple groups. We performed statistical analysis using SPSS software version 26.0 (IBM Corp., Armonk, NY, USA). *P < *0.05 was considered statistically significant.
