# Supplementary material for: α-Mangostin Exhibits a Therapeutic Effect on Spinal Cystic Echinococcosis by Affecting Glutamine Metabolism
Source: Antimicrob Agents Chemother. 2023 May 4;67(6):e00098-23. doi: 10.1128/aac.00098-23 (PMC10269084; doi:10.1128/aac.00098-23)
Supplement: Supplemental file 1 — Figure S1 to S3. Download aac.00098-23-s0001.pdf, PDF file, 1.64 MB [file aac.00098-23-s0001.pdf]

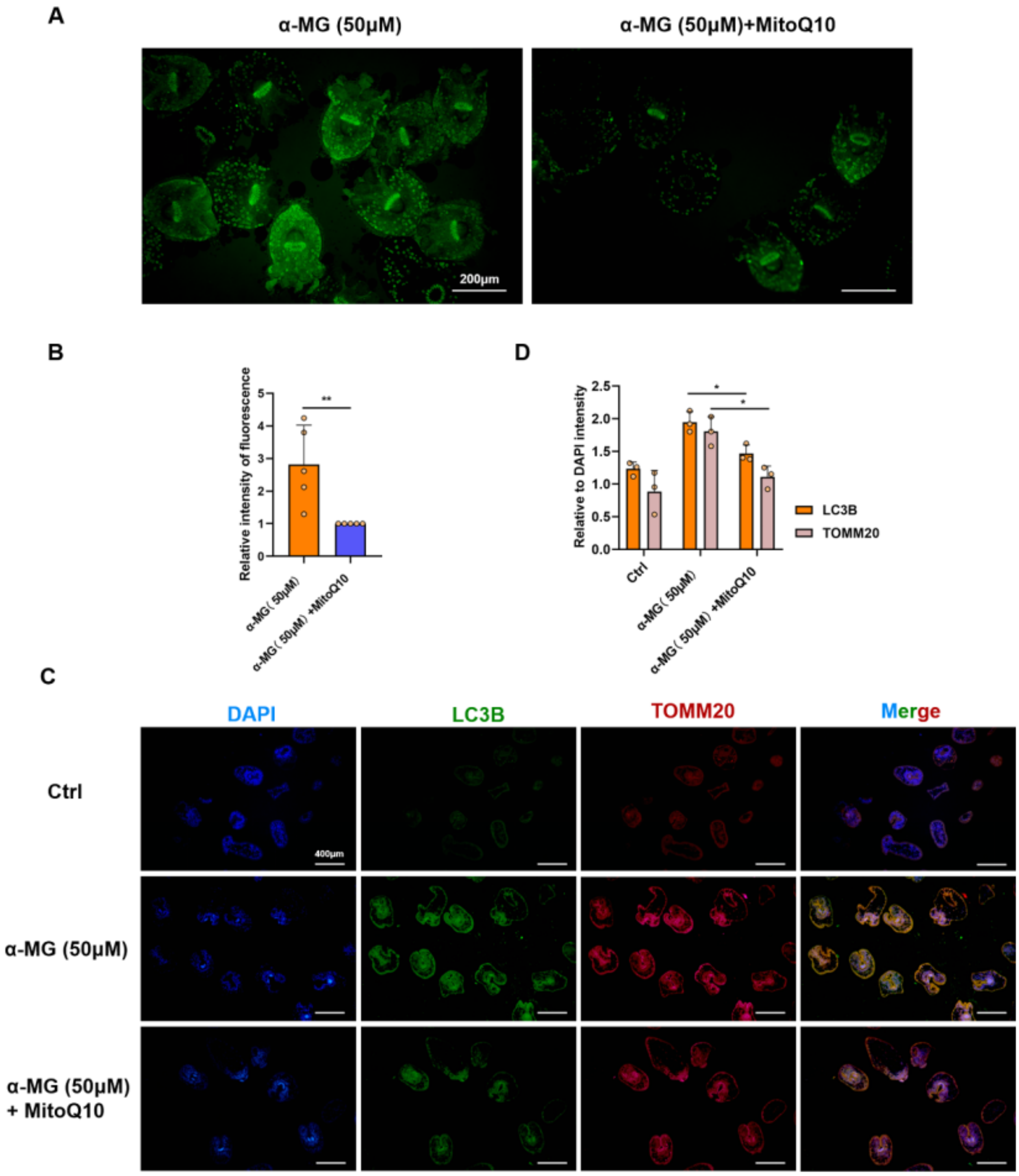

FIG S1 α-MG-induced autophagy is dependent on ROS generation. (A) Representative images showing ROS fluorescence in PSCs incubated 3 days with 50 μM of α-MG combined with 10 μM of MitoQ10 (n = 5). (B) Quantitative analysis of relative ROS fluorescence intensity in different treatment groups. (C) Representative immunofluorescence images of PSCs cells treated with α-MG and in combination with MitoQ10. (D) The ratio of LC3B and TOMM20 proteins to DAPI fluorescence intensity was shown (n=3). Data are presented as mean ± SD. * $P < 0.05$; ** $P < 0.01$ using the two-sided Student's-t test.

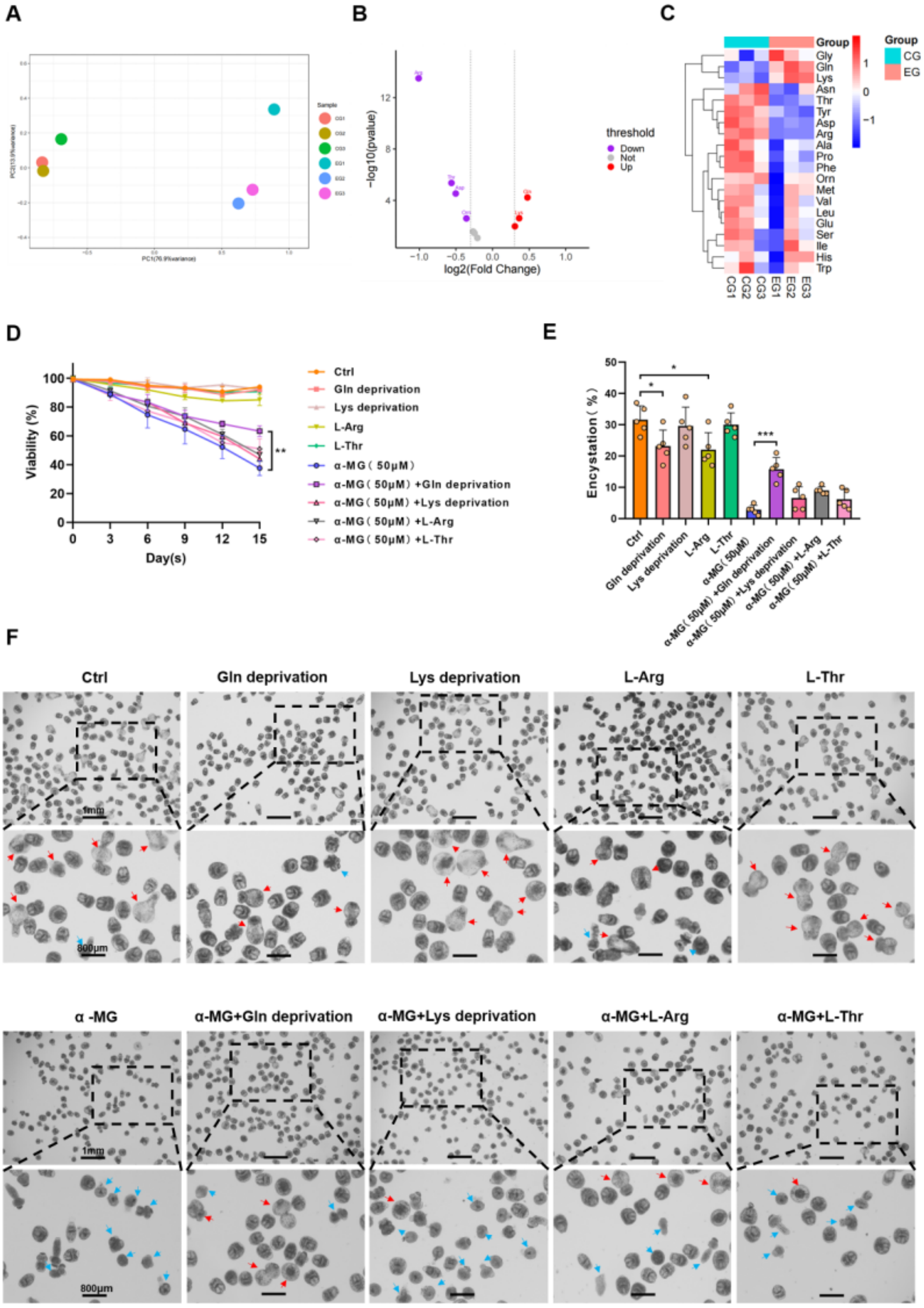

FIG S2 Glutamine deficiency is essential for the antiparasitic effect of the α-MG. (A) The score plot of PCA shows a clear difference between the samples from the drug-treated (50 μM) and control groups. CG: control group; EG: experimental group. (B) Volcano plot of amino acid concentration in PSCs. (C) Z-score clustering analysis between two groups. Each square represents the clustering value of amino acids in the samples within the

cluster. Warm (red) or cool (blue) colors indicate elevated or decreased metabolites between the two groups on the horizontal axis. (D) PSCs was cultured in amino acid conditioned medium with or without α-MG (50μM) for 15 days (n=3). (E) The data represent the evolution rate of PSCs in different conditioned medium in vitro (n=5). (F) PSCs were cultured in different amino acid conditioned medium for 15 days to observe in vitro evolution. Red arrows indicate encysted larvae, blue arrows indicate dead larvae. Data are the mean ± SD. * $P < 0.05$; ** $P < 0.01$; ***, $P<0.001$ using the two-sided Student's-t test.

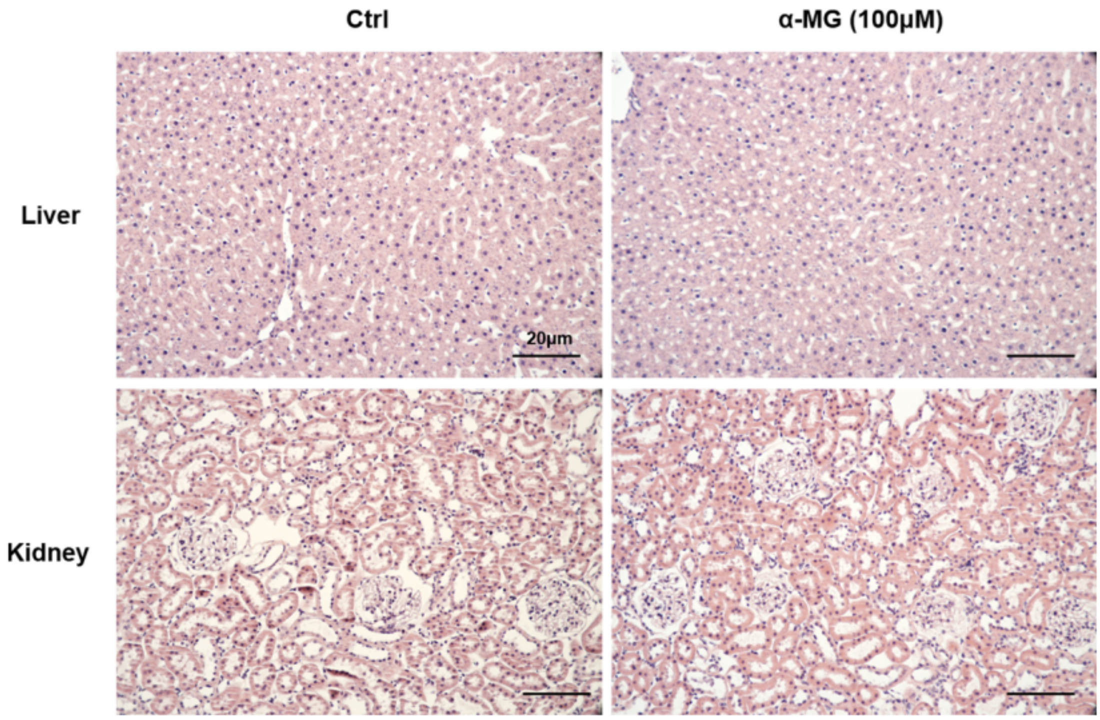

FIG S3 Toxicity of α-MG in vivo. The toxicity of the α-MG treatment was assessed by morphological observation of liver and kidney tissues in vivo. HE stained images showed no significant pathological changes or damage to liver and kidney tissues.
